# Breakdown of albumin and haemalbumin by the cysteine protease interpain A, an albuminase of *Prevotella intermedia*

**DOI:** 10.1186/s12866-015-0516-3

**Published:** 2015-09-24

**Authors:** Dominic P. Byrne, Surya P. Manandhar, Jan Potempa, John W. Smalley

**Affiliations:** The University of Liverpool, School of Dentistry, Daulby Street, Liverpool, L69 3GN UK; Department of Biochemistry, Institute of Integrative Biology, The University of Liverpool, Crown Street, Liverpool, L69 7ZB UK; Department of Biological Sciences, California State University Long Beach, 1250 Bellflower Blvd., Long Beach, California 90840 USA; Malopolska Centre of Biotechnology and Department of Microbiology, Faculty of Biochemistry, Biophysics and Biotechnology, Jagiellonian University, Gronostajowa 7, Krakow, 30-387 Poland; Department of Oral Immunology and Infectious Diseases, University of Louisville School of Dentistry, 501 S. Preston St., Louisville, KY 40202 USA

**Keywords:** Albumin, Protease, Periodontal disease, Haem, Interpain A, *Prevotella intermedia*

## Abstract

**Background:**

*Prevotella intermedia* is a Gram-negative black-pigmenting oral anaerobe associated with periodontitis in humans, and has a haem requirement for growth, survival and virulence. It produces an iron porphyrin-containing pigment comprising monomeric iron (III) protoporphyrin IX (Fe(III)PPIX.OH; haematin). The bacterium expresses a 90-kDa cysteine protease termed interpain A (InpA) which both oxidizes and subsequently degrades haemoglobin, releasing haem. However, it is not known whether the enzyme may play a role in degrading other haem-carrying plasma proteins present in the gingival sulcus or periodontal pocket from which to derive haem. This study evaluated the ability of InpA to degrade apo- and haem-complexed albumin.

**Results:**

Albumin breakdown was examined over a range of pH and in the presence of reducing agent; conditions which prevail in sub- and supra-gingival plaque. InpA digested haemalbumin more efficiently than apoalbumin, especially under reducing conditions at pH 7.5. Under these conditions InpA was able to substantially degrade the albumin component of whole human plasma.

**Conclusions:**

The data point to InpA as an efficient “albuminase” with the ability to degrade the minor fraction of haem-bound albumin in plasma. InpA may thus contribute significantly to haem acquisition by *P. intermedia* under conditions of low redox potential and higher pH in the inflamed gingival crevice and diseased periodontal pocket where haem availability is tightly controlled by the host.

## Background

Members of the genus *Prevotella* are associated with periodontal diseases in humans and other animals [1]. They have a growth requirement for iron protoporphyrin IX, and display a characteristic black-pigmenting phenotype due to cell-surface accumulation of haem (iron(III) protoporphyrin IX) in the monomeric form (Fe(III)PPIX.OH, haematin), derived *via* the breakdown of haemoglobin [2]. The haem-containing pigments of both *Prevotella* and *Porphyromonas* species are important defensively, since ferrihaems in both the monomeric and dimeric ([Fe(III)PPIX]_2_O) forms possess inherent catalase activity and thus can protect against hydrogen peroxide [3] which is produced by activated neutrophils.

In the primary habitats of the black-pigmenting anaerobes (the inflamed gingival sulcus and periodontal pocket), the most abundant haem source is haemoglobin. However, in healthy or minimally inflamed gingival crevices, in the absence of overt bleeding (and hence haemoglobin), their survival and growth may depend on haemalbumin and haem-haemopexin as primary haem sources. These proteins normally sequester any circulating free haem and transport it to the liver [4, 5], thus limiting its bioavailability to invading micro-organisms.

Albumin is the most predominant protein from both healthy and inflamed periodontal disease sites [6–8]. Although albumin has a 75-fold lower affinity for haem compared to haemopexin [9], its haem sequestering capability is compensated by its 40-80-fold greater concentration in serum [10], and therefore it binds the majority of any haem liberated into the circulation. In addition, in normal serum, approximately 1 in 5000 albumin molecules is haem liganded [11]. Importantly, these haemalbumin molecules may serve as a non-haemoglobin source of iron protoporphyrin IX under conditions of haem limitation for *P. intermedia* and other sub- and supra-gingival plaque bacteria.

We have investigated the mechanism of protease-mediated haem release from haemoglobin by *P. intermedia* [12]. This organism expresses a 90-kDa cysteine protease, termed interpain A (InpA) [13], an orthologue of periodontain (PrtP) of *P. gingivalis* and streptopain (SpeB) of *Streptococcus pyogenes* [14, 15]. InpA is likely to serve a major role in pigment production through oxidising oxyhaemoglobin and then fully degrading the methaemoglobin product to release haem [12]. In addition, it has been recently shown that InpA may serve to aid *P. gingivalis* haem acquisition by proteolytically mediating formation of methaemoglobin from which the *P. gingivalis* HmuY haemophore can extract haem [16]. Cell associated protease activity of *P. intermedia* can degrade albumin and haemopexin [17, 18], although the proteases mediating this have not been characterised. Incubation of the bacterium with albumin intensifies its proteolytic activity and growth [19]. Given that albumin represents an important source of haem under conditions of haem scarcity, we examined the ability of InpA to degrade albumin in both its apo- and haem-liganded forms under conditions which may prevail in the primary habitats of this bacterium, the inflamed gingival sulcus and diseased periodontal pocket.

## Results and discussion

**Fig. 1:**
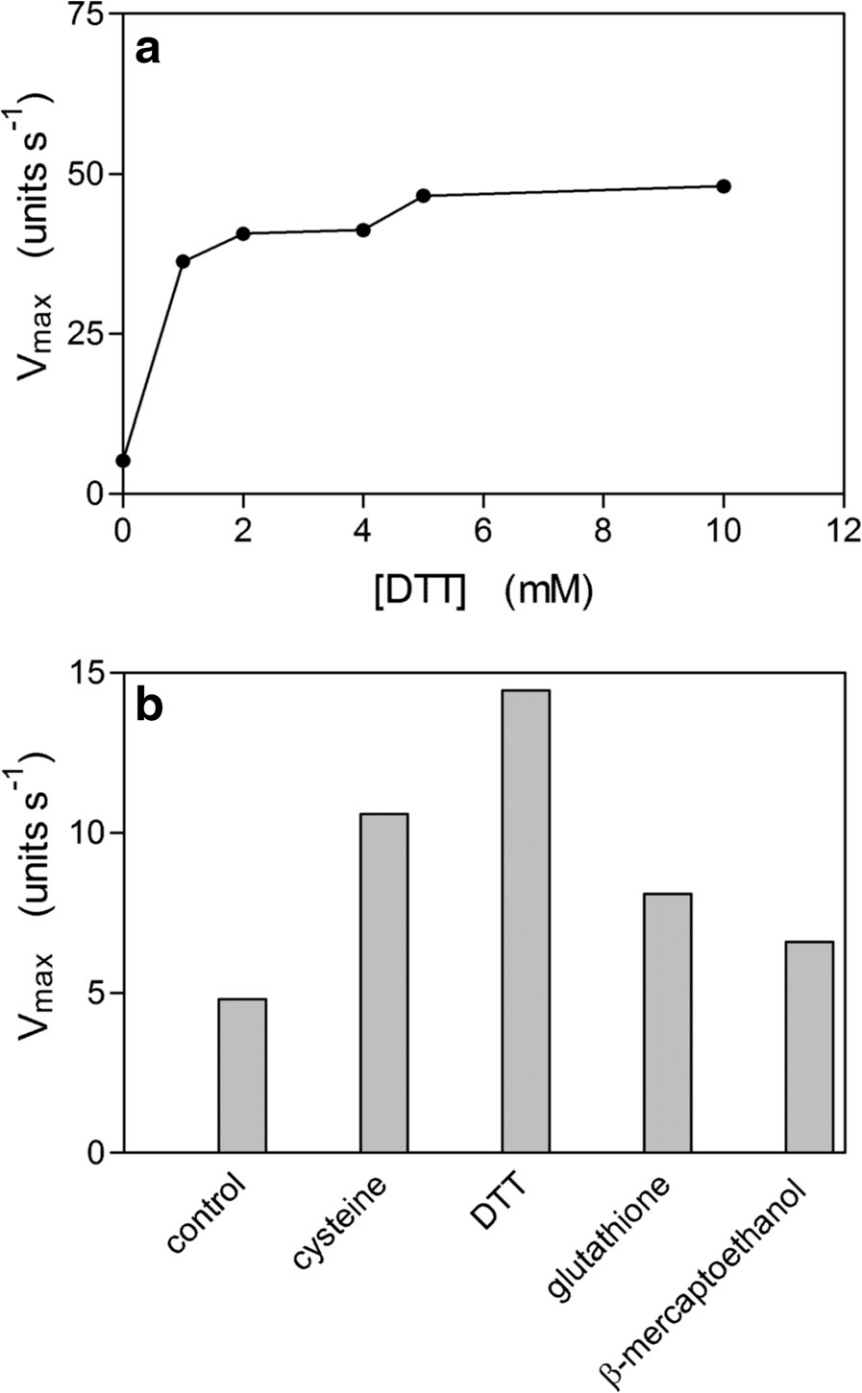
Effect of dithiothreitol (**a**) and other thiol reducing agents (**b**) on the enzyme activity of InpA. The enzyme activity was assayed in triplicate using Z-Arg-Arg-AMC at 37 °C in 0.1M Tris–HCl, pH 7.5 and the concentrations of the reducing agents in Fig. 1b were 3 mM. Due to the high reproducibility of the replicates, no SD is apparent. Control, activity in the absence of reducing agent. InpA concentrations in (**a**) and (**b**) were 12.5 μg ml^−1^. See text for details

Since the redox potential of diseased periodontal pockets can be as low as −300 mV [20] and that *P. intermedia* may experience wide fluctuations of pH within dental plaque, it was considered appropriate to examine the effect of these two variables on the activity of InpA. Our previous study [13] showed that InpA activity was stimulated by dithiothreitol (DTT) and so we examined the effect of various concentrations of DTT on InpA activity versus the fluorescent synthetic substrate Z-Arg-Arg-AMC (250 μM). This revealed that enzyme activity began to plateau at 2 mM (Fig. 1a). We then determined the influence of the thiol reducing agents cysteine, reduced glutathione and β-mercaptoethanol (all at 2 mM) on InpA activity using the above substrate, and this showed that DTT gave the greatest stimulatory effect (Fig. 1b). However, in subsequent experiments to examine the influence of reducing conditions on albumin and haemalbumin degradation, we employed 3 mM DTT to ensure full enzyme activation.

**Fig. 2:**
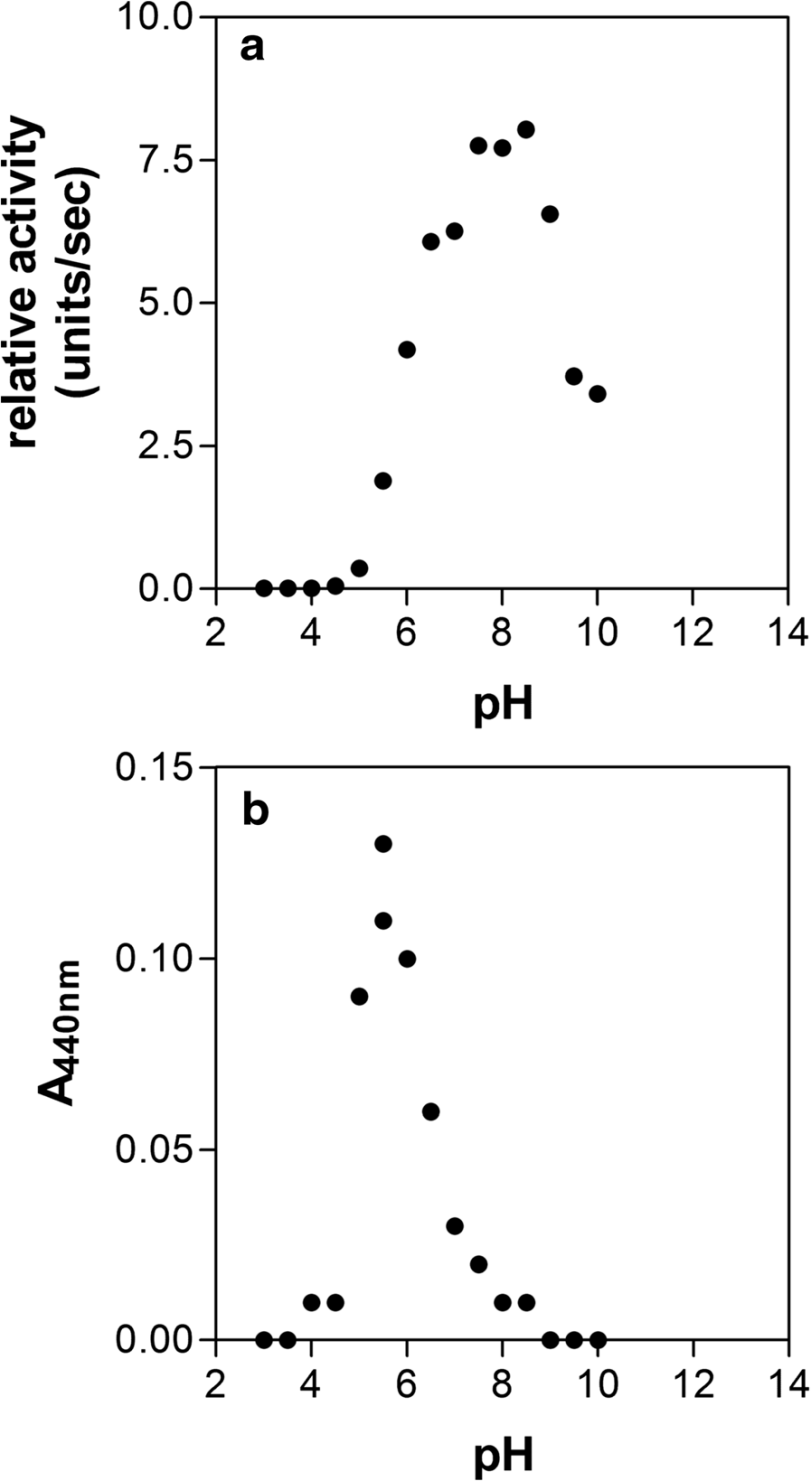
Effect of pH on InpA activity towards Z-Arg-Arg-AMC (**a**) and azoalbumin (**b**). Protease activity was assayed at 37 °C in PBS, pH 7.4, containing 3 mM DTT. InpA concentration was 50 μg ml^−1^. Azoalbumin concentration was 3 % (w/v). Enzyme activity is depicted as absorbance of azo dye (A_440_) released from the protein. See text for details

InpA displayed a broad activity against Z-Arg-Arg-AMC over the pH range from 6 to 10 (Fig. 2a). However, for azo-albumin, the maximal activity was centred at pH 5.5, with little or no activity beyond pH 4 or above 8 (Fig. 2b). This may be important in terms of protein breakdown since *P. intermedia* can generate acid during growth in both liquid and solid media [2, 21, 22], and may thus depress the pH locally *in vivo*. We also investigated the temperature activity profile for InpA and thermal stability of the enzyme using azocoll and the synthetic substrate Z-Arg-Arg-AMC, respectively. The enzyme showed maximal activity in the temperature range from 30 to 40 °C versus azocoll (data not shown). However, the enzymatic activity was found to be drastically decreased after the protein was pre-incubated for 30 min at temperatures above 50 °C, followed by assay with Z-Arg-Arg-AMC (data not shown). This indicates that InpA should be highly active at elevated temperatures which may be encountered *in vivo* in the inflamed gingival sulcus or periodontal pocket during periods of inflammation. .

**Fig. 3:**
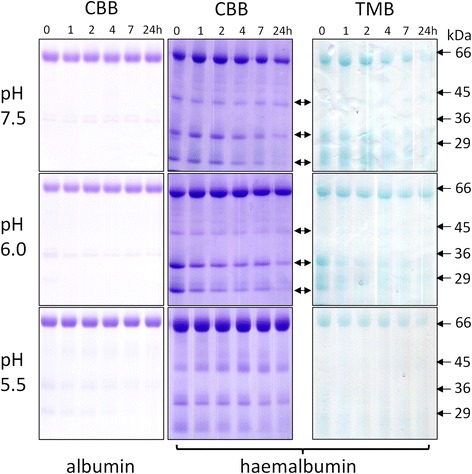
Albumin and haemalbumin breakdown by InpA at different pH under non-reducing conditions. Albumin and haemalbumin (1:1 haem:protein ratio) (16 μM) were incubated with InpA (100 μg ml^−1^) at 37 °C and aliquots of the incubations were sampled periodically at 1, 2, 4, 7 and 24 h and InpA activity inhibited by E-64 (0.5 mM) and subjected to SDS-PAGE on 10 % non-reducing gels. Samples were solubilised at 37 °C for 1 h in non-reducing application buffer. The haemalbumin gels were stained with TMB to reveal haem-associated peroxidise activity and counterstained with CBB for protein, whilst albumin gels were stained only with CBB. Protein loading was ≈ 20 μg per lane. Arrows denote digestion fragments carrying bound haem

In the absence of DTT, there was little or no degradation of albumin (Fig. 3), and densitometry showed that total apoalbumin degradation over 24 h was less than 5 % at all three pHs studied. In contrast however, for haemalbumin, three lower molecular weight peptide fragments of R_f_ 0.68, 0.83 and 0.94, were generated at pH 7.5 and 6.0 (arrowed). The two fastest migrating of these bands, which stained with TMB-H_2_O_2_ and hence retained bound haem, were further degraded with time and also showed progressive loss of haem staining, as did the main 66-kDa haemalbumin band, especially at pH 7.5 after 4 h. Little protein degradation was observed at pH 5.5. However, after 24 h, TMB-H_2_O_2_ staining of haemalbumin had decreased by circa 45 % at all pHs examined, showing that extensive proteolysis was not necessary to effect haem release from the molecule. In contrast however, the percentage of haemalbumin protein degraded after 24 h was greater at pH 7.5 (35 % ± SD 13), compared to either pH 6.0 (20 % ± SD 6) or pH 5.5 (4 % ± SD 6) (*n* = 4 in all cases). It should be noted that both apo- and haemalbumin were stable over 24 h at all pH conditions tested in the absence of InpA (data not shown).

Despite InpA displaying optimum activity towards azo-albumin at pH 5.5 (Fig. 2) and being more active against aquomethaemoglobin at this pH [12], it is interesting that haemalbumin degradation by InpA was negligible at pH 5.5 but greatest at the slightly alkaline pH of 7.5. This apparent increase may not reside in InpA activity *per se*, but may result from structural changes in the albumin molecule occurring in solution at high pH [23, 24]. Albumin displays a greater thermal stability at pH 6.4 than at pH 7.4 [25], undergoing a conformational change around pH 7.5 referred to as the N → B transition [26], pointing to the B form being more susceptible to InpA, as has been demonstrated for trypsin [27]. This may be physiologically relevant as diseased periodontal pockets and the inflamed gingival sulcus are slightly alkaline [28, 29, 30]. However, SDS-PAGE revealed (Fig. 3) that haem loss also occurred at acid pH despite the low level of proteolysis, suggesting that only limited proteolysis by InpA is required to loosen the haem-albumin association.

Compared to apoalbumin, haemalbumin is more susceptible to InpA. Haem complexing to bovine serum albumin also leads to greater proteolysis by trypsin and chymotrypsin [30] although proteolysis of haemalbumin by whole cells of *P. gingivalis* is decreased, particularly as the ratio of bound haem to protein increases [31]. Nevertheless, conformational changes occurring upon haem ligation [11] may account for InpA attack, enabling InpA to differentiate between apoalbumin and the scarcer haem-liganded form, which represents approximately 1/5000 of the total albumin molecules in serum under normal physiological conditions [12, 33].

**Fig. 4:**
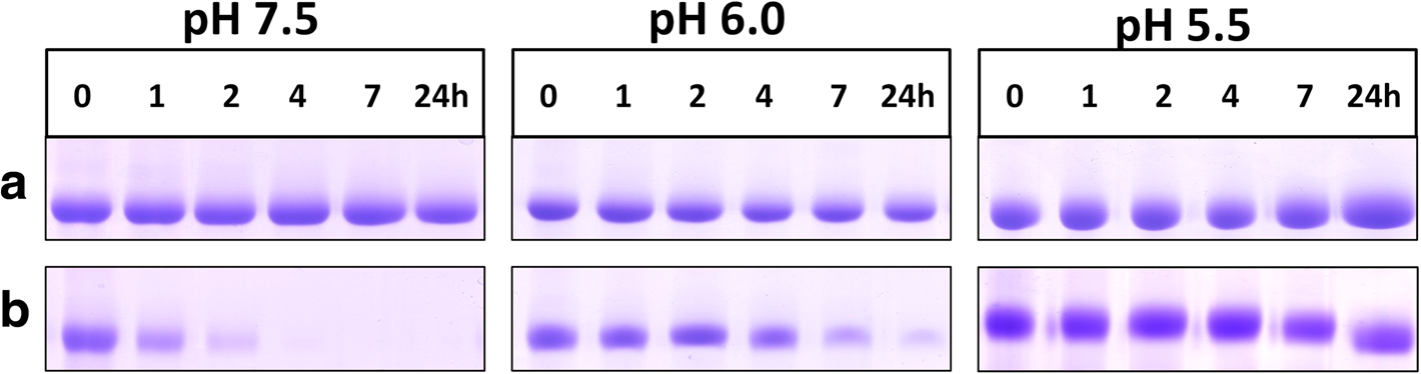
Chemical reduction increases the susceptibility of albumin to InpA. Albumin (16 μM) was incubated with InpA (100 μg ml^−1^) at different pH at 37 °C under non-reducing (**a** panels) and reducing conditions (3 mM DTT) (**b** panels). Gel lanes had a nominal loading of 20 μg albumin, and are stained with CBB. Samples were prepared and gels run as described for Fig. 3

**Fig. 5:**
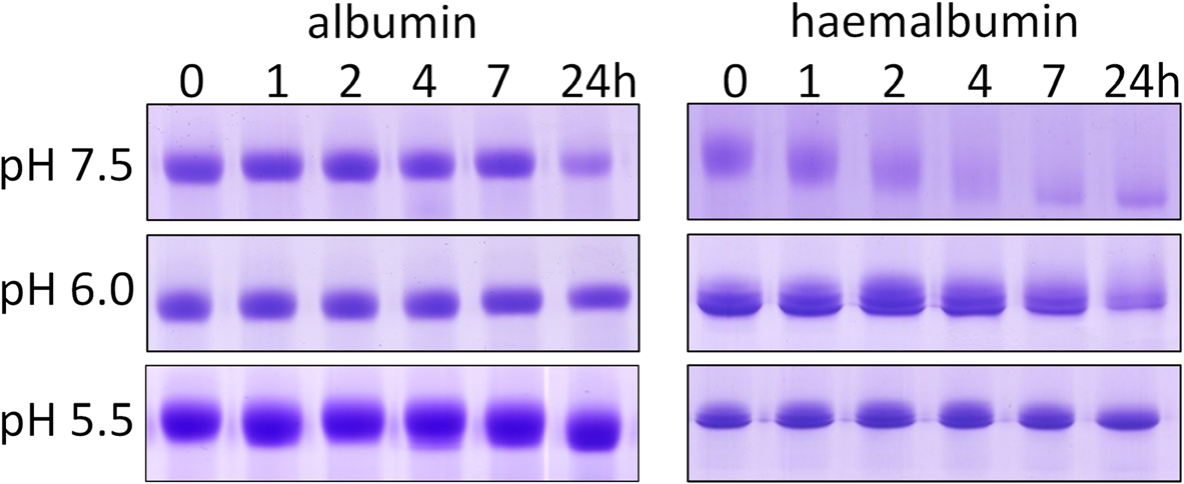
SDS-PAGE showing the stability of albumin and haemalbumin under reducing conditions at different pH. Albumin and haemalbumin (16 μM) were reduced with 3 mM DTT and incubated at 37 °C. SDS-PAGE conditions were the same as those in Fig. 3. The gels were stained with CBB

In stark contrast to non-reducing conditions, albumin degradation was greatly increased under reducing conditions (3 mM DTT) at all pHs examined, but especially at pH 7.5, where complete breakdown was observed after 4 h (Fig. 4). The presence of DTT also increased albumin susceptibility at pH 6.0 (86 % after 24 h). However, at pH 5.5 less than 10 % was broken down even when DTT was present. Albumin was found to be stable under reducing conditions in the absence of InpA as judged by minimal depreciation in CBB staining observed only after 24 h incubation at pH 7.5 (Fig. 5). In contrast to reducing conditions, apoalbumin was less susceptible to InpA (Fig. 3), and densitometric scans (data not shown) indicated that approximately 20 % had been degraded after 7 h at each pH.

**Fig. 6:**
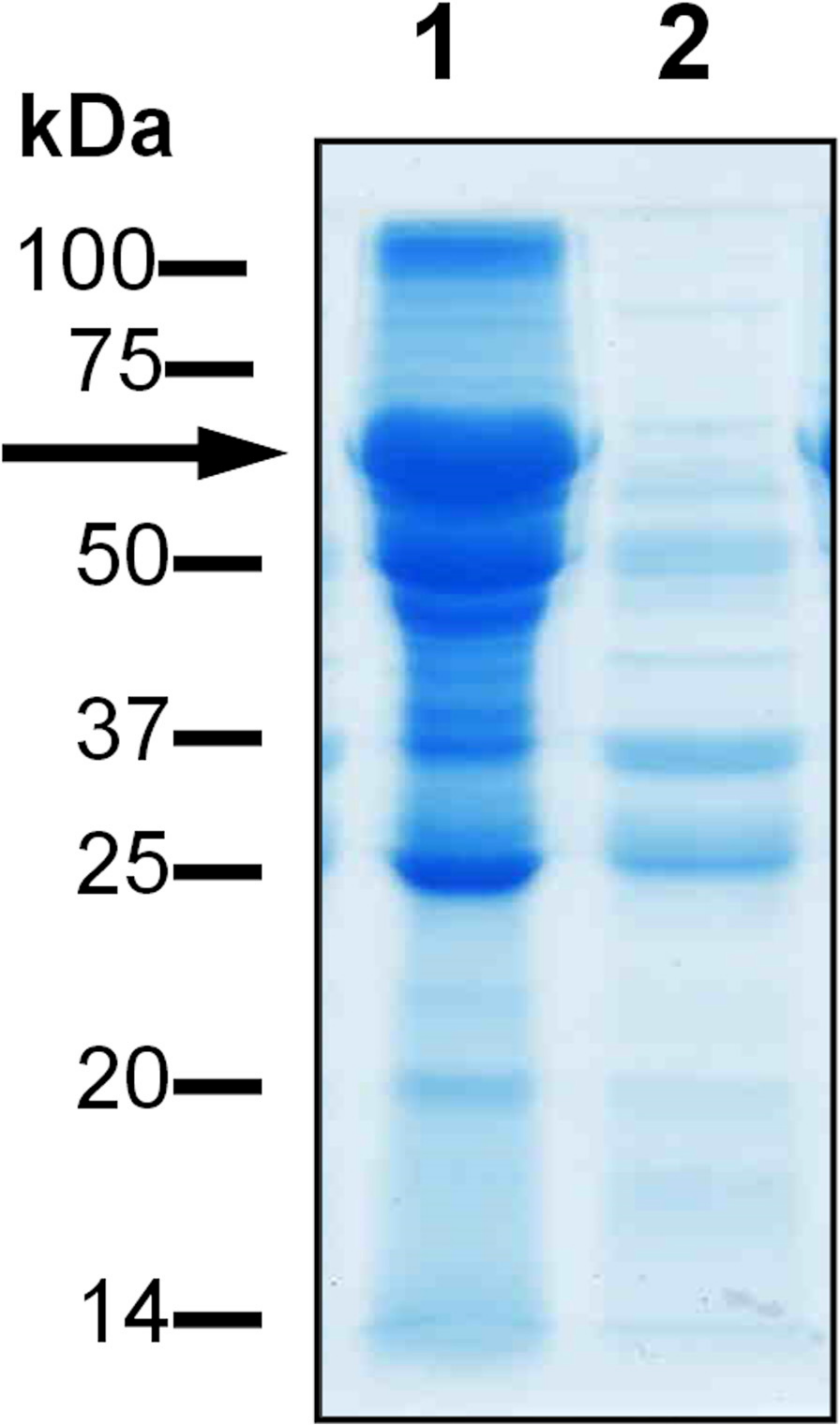
SDS-PAGE analysis of the breakdown of whole human plasma by InpA. Plasma (1 μl, ~60μg protein), was incubated overnight at 37 °C in PBS, pH 7.4, containing 3 mM DTT in absence (lane 1) or presence (lane 2) of active InpA (100 μg ml^−1^) in a final volume of 20 μl. ~ 50 μg total protein was loaded per lane. Arrow denotes albumin band. Samples were solubilised for 5 min at 100 °C in reducing application buffer, and gels were stained with CBB

**Fig. 7:**
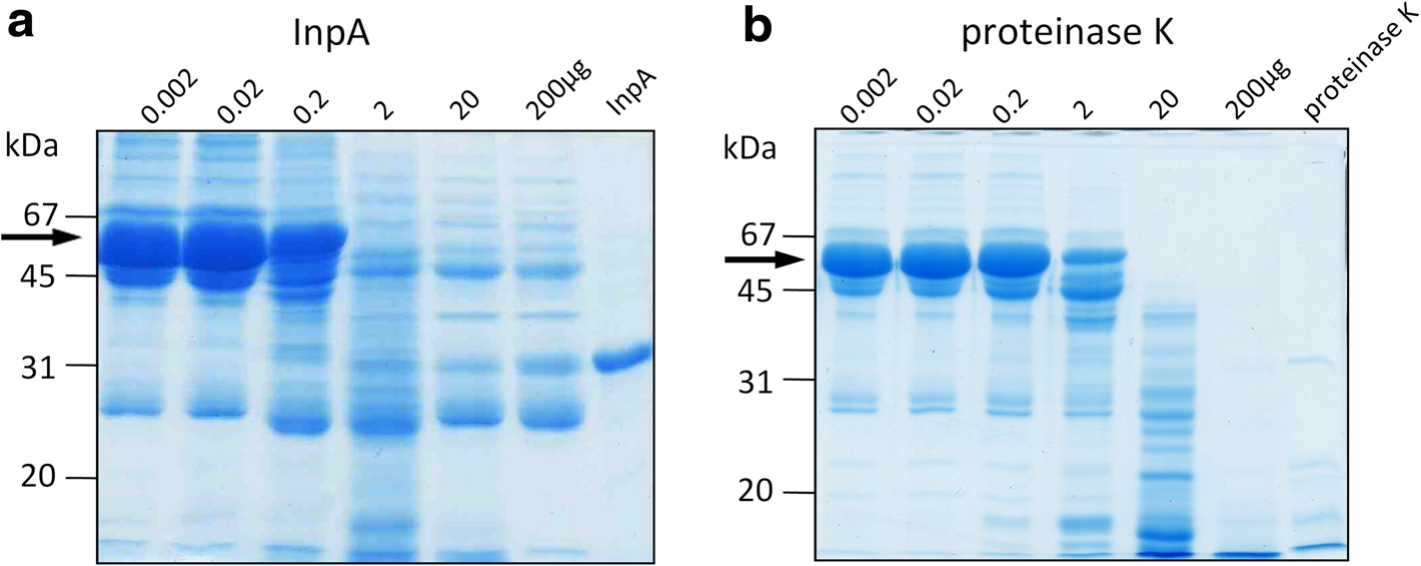
Comparative SDS-PAGE analysis of the digestion of whole human plasma by InpA and proteinase K. Whole human plasma (60 μg total protein) was incubated overnight with InpA and proteinase K (Sigma-Aldrich; product number P2308) at the indicated concentrations (μg ml^−1^) at 37 °C in PBS, pH 7.4 containing 3 mM DTT. Last lane of each gel was loaded with 10 μg of either InpA or proteinase K only. Samples were prepared and gels stained as in Fig. 6

In view the above, and the compositional similarities between gingival crevicular fluid and plasma [6], it was appropriate to examine the effect of InpA on whole blood plasma, under conditions commonly reflecting those in the inflamed sulcus and periodontal pocket i.e., alkaline pH and low redox potential. SDS-PAGE revealed that overnight incubation of whole human plasma with InpA at pH 7.5 and in the presence of 3 mM DTT, resulted in almost complete breakdown of the albumin band (Fig. 6a, arrowed), whilst leaving many other plasma proteins relatively undigested. In comparison, proteinase K, a broad spectrum serine endopeptidase without any peptide bond preference, degraded a large number of the proteins in whole plasma but was less efficient at degrading the albumin component when compared to InpA at the same enzyme concentration (Fig. 7a and b, arrowed). These data show that InpA is able to target albumin present in plasma.

**Fig. 8:**
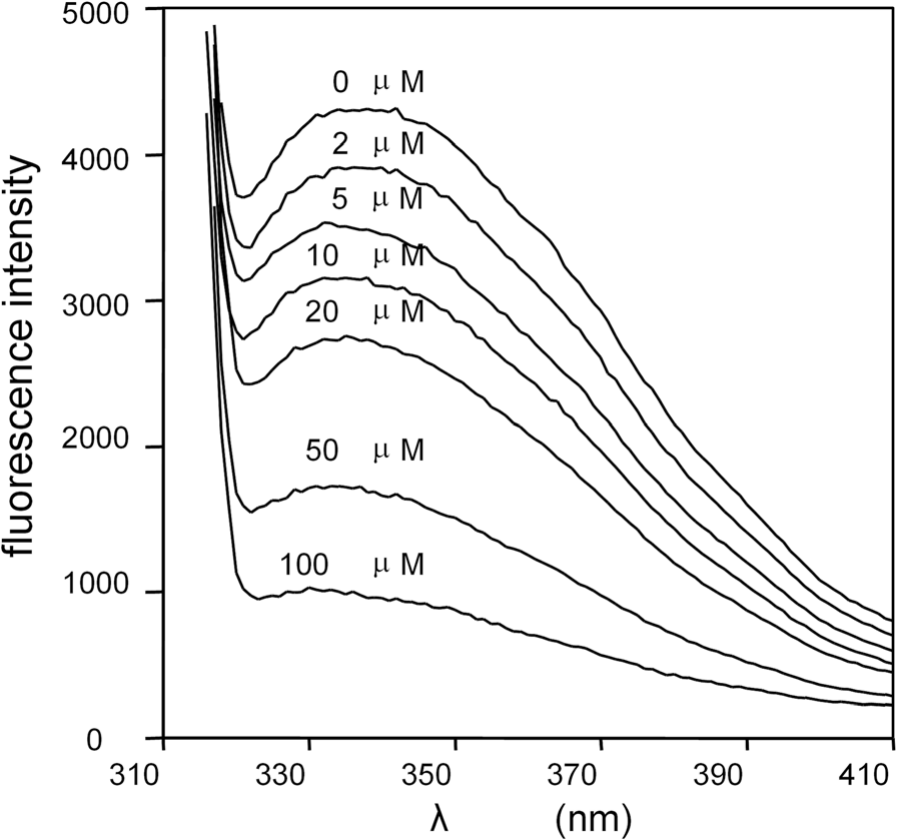
Intrinsic fluorescence spectra of bovine albumin in the presence of DTT. Albumin (16 μM) was treated with increasing concentrations of DTT in 0.14 M NaCl, 0.1 M Tris–HCl buffer, pH 7.5, at 37 °C, and the fluorescence emission monitored after excitation at 295 nm

The increased susceptibility of albumin to InpA in the presence of DTT may arise from structural changes brought about by chemical reduction of the disulphide bridges. Conformational changes of the local tryptophan environment in albumin are reflected in fluctuations of intrinsic fluorescence [32, 33]. When apoalbumin was treated with DTT there was a reduction of fluorescence intensity and moderate shift of λ_max_, indicating a change in the protein structure (Fig. 8). Fluorescent emission was not detected above 100 μM DTT, indicating that the concentrations used in the digestions (2–10 mM) were sufficient to induce structural alterations in albumin. Since InpA was pre-activated with an excess of DTT, it is likely that this increased susceptibility of albumin to InpA under reducing conditions was due to structural changes in the substrate rather than to any over-stimulatory effect of DTT on protease activity *per se*.

The low redox potential of diseased periodontal pockets is attributable to the presence of volatile sulphur compounds (VSCs) including H_2_S and methyl mercaptan, generated by organisms including *P. intermedia*, *P. gingivalis* and *Fusobacterium nucleatum* [34, 35]. *P. intermedia* is also recovered from periodontal disease sites of patients suffering from oral malodour linked to VSC production [36]. The 17 disulphide bridges of albumin are sensitive to reduction, which stimulates tertiary structural changes [33, 37], leading to increased molecular flexibility and enhanced susceptibility to pepsin [38]. Chemical reduction resulting from bacterial metabolism occurs during batch-wise enrichment culture of sub-gingival plaque in serum, where redox potentials of -70 to −550 mV have been recorded, and correlate with both H_2_S production and enrichment in numbers of *P. intermedia* [39]. Since 3 mM DTT was sufficient for optimal InpA activity, and is a concentration which can depress the redox potential to between −60 and −220 mV in *E. coli* growth medium [40], it is likely that the reducing environment of deep periodontal pockets also results in structural changes in albumin because of disulphide bond disruption [33], rendering albumin highly susceptible to InpA. Predictably therefore, treatment of albumin with 3 mM DTT greatly increased degradation by InpA, especially at pH 7.5. The minimal breakdown at acid pH with DTT present, where InpA activity towards azo-albumin and aquomethaemoglobin is highest [12], suggests that pH-dependent conformational changes [23, 25, 41] may dictate the effectiveness of proteolysis by InpA. It is noteworthy also that accessibility of reducing agents to disulfide bonds in albumin (which would be in the B form) increases above pH 7.0 [42].

**Fig. 9:**
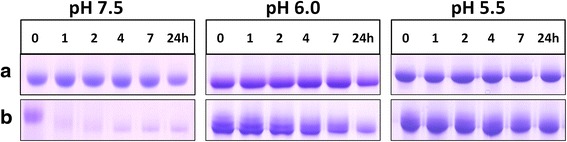
Effect of reducing conditions on breakdown of haemalbumin by InpA at different pH. Haemalbumin (16 μM) with a 1:1 haem to protein ratio was incubated at 37 °C with InpA (100 μg ml^−1^) in the absence (**a** panels) and presence of 3 mM DTT (**b** panels). Samples and gels were run under non-reducing conditions on 10 % gels and stained for protein with CBB. Each gel lane had a nominal loading of 20 μg albumin

In the presence of 3 mM DTT the susceptibility of haemalbumin (1:1 haem: protein ratio) to InpA was also dramatically increased (Fig. 9). This was especially noticeable at pH 7.5; an observation explained by the fact that haemin binding to albumin expands sub-domain IB (normally constrained by the Cys124 and Cys169 S-S bridges [43]) which may render other regions of the peptide chain available for attack. To further assess the ability of InpA to bring about proteolytic haem release from albumin at pH 7.5 haemalbumin conjugated agarose was used as a substrate. After 1 h in the presence of DTT, InpA liberated approximately 3-fold more haem than under non-reducing conditions (data not presented). In this context it is noteworthy that the albumin-haem affinity is lowered under reducing conditions, which *in vivo* favours haem transfer to haemopexin [44]. Furthermore, we observed that under reducing conditions haemalbumin was intrinsically more unstable than its non-liganded counterpart, particularly at alkaline pH (Fig. 5), which may explain its heightened susceptibility to enzymic digestion. Thus, should the local plaque or periodontal pocket redox potential decline, the additional effect of reducing agents in facilitating haem liberation may supplement InpA-mediated haem release.

It is noteworthy that Grenier *et al.* [45] demonstrated albumin breakdown by *P. gingivalis* R- and K-gingipains in the presence of 10 mM DTT. We have observed that albumin and haemalbumin are resistant to active forms of both HRgpA and Kgp under non-reducing conditions (data not presented). These and the above findings highlight the importance of reducing conditions which prevail in the diseased periodontal pocket and which are essential for disrupting both the haem liganded and apoalbumin structures.

**Fig. 10:**
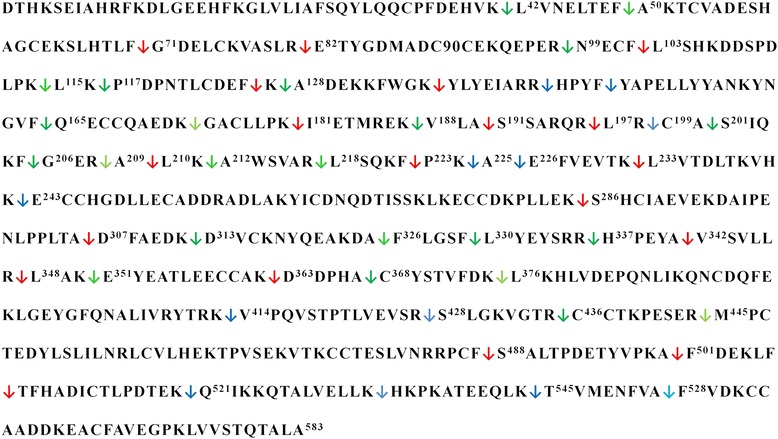
Primary sequence showing all the cleavage sites inflicted during incubation of bovine serum albumin with InpA. Albumin (16 μM) was incubated at 37 °C with InpA (100 μg ml^−1^) in 10 mM Tris buffer, pH 7.5, in the presence or absence of 2mM DTT, and the incubation mixture sampled over time subjected to MALDI-TOF mass spectrometry. InpA scission sites exclusive to reducing () , non-reducing (), and to both incubation conditions () are indicated. See text for details

InpA has specificity for K, R, A and F residues at the P1 position of hydrolysed peptide bonds [12; J. Potempa, unpublished findings] and this was seen in the large number of peptide fragments generated under both reducing and non-reducing conditions (Fig. 10). In all, 19 InpA scission sites were exclusive to reducing conditions and 12 to non-reducing conditions, with 24 sites common to digestions under both incubation conditions. The formation of more fragments in the presence of DTT is not surprising, as it may reflect the exposure of more susceptible cleavage sites at previously inaccessible residues resulting from disulphide bond disruption.

Bleeding, and consequently increased levels of haemoglobin and hence haem, is associated clinically with the inflamed gingival crevice and diseased periodontal pocket, where the host responds to bacterial presence by increasing levels of plasma stress proteins including haemopexin and albumin [6, 46, 47]. In addition, the combined haemolytic and proteolytic activities of other sub-gingival micro-organisms may also transiently elevate local haem levels resulting in greater haem-albumin concentrations. However, we show that InpA not only degrades apoalbumin which normally reduces the potential for haem-induced inflammatory events [48] and limits the bioavailability of haem to micro-organisms, but is more active towards the haem-liganded protein. Depreciation in haem scavenging by albumin at periodontal sites through the action of InpA would hinder local haem clearance and potentially advantage *P. intermedia* and other haem-requiring organisms. In addition, in regard to the periodontal pocket environment, it is known that in addition to low redox potential, slightly alkaline pH conditions also severely perturb host haem recycling, since they lower the albumin-haem binding affinity [44, 49].

## Conclusions

The current findings show that InpA can function as an albuminase and may enable *P. intermedia* to specifically target albumin present in whole plasma. Although reducing and slightly alkaline conditions may be optimal for InpA breakdown of albumin and haem release, this protease may also be versatile in degrading albumin in a plaque environment where non-reducing conditions prevail, such as a newly inflamed site or at early stages of periodontal pocket formation. In conclusion, by more easily degrading haem-liganded albumin, InpA may contribute to haem acquisition by *P. intermedia* where the availability of this essential growth factor is otherwise tightly restricted by the host.

## Methods

### Purification of InpA

InpA was produced as a recombinant protein in *Escherichia coli* and purified as previously described [12, 13] by affinity chromatography on Fast Flow Ni-NTA (Ni^2+^-nitrilotriacetate)–Sepharose (Qiagen) followed by anion exchange chromatography (MonoQ, GE Healthcare). The active concentration of InpA was determined by active-site titration using an appropriate dilution of a standardized 1 mM aqueous solution of the protease inhibitor N-(*trans*-epoxysuccinyl)-L-leucine 4-guanidinobutylamide (E-64) (Sigma-Aldrich; product E3132) needed for total inactivation of the proteinase. Residual enzyme activity was determined by measurement of fluorescence (λ_ex_ = 380 nm and λ_em_ = 460 nm) of AMC (7-amino-4-methylcoumarin) released from t-butoxycarbonyl-Val-Leu-Lys-AMC as described previously [13]. Immediately before use, InpA was activated by incubation for 15 min with 3 mM DTT in 0.1 M Tris–HCl, 0.14 M NaCl, pH 7.5 [50]. When used for protein breakdown assays under non-reducing conditions, the above buffer was exchanged for that without reducing agent by ultrafiltration using 10 kDa cut-off Microcons (Amicon).

### Gingipain purification

Soluble HRgpA and Kgp gingipains were purified from the culture medium of *P. gingivalis* strain HG66 as described previously using gel filtration and arginine–Sepharose chromatography [51, 52]. Before use, both HRgpA and Kgp were also activated for 15 min in 0.1 M Tris–HCl, 0.14 M NaCl, pH 7.5, plus 3 mM DTT. However, when employed to determine protein degradation in non-reducing conditions, this buffer was replaced for that without DTT by ultrafiltration as above.

### Effect of thiol reducing agents on InpA activity

The fluorescent substrate Z-Arg-Arg-7-amido-4-methylcoumarin (Z-Arg-Arg-AMC; 250 μM) was used to determine the enzyme *V*_*max*_ as previously described [51], the hydrolysis of AMC being recorded by measuring fluorescence increase (λ_ex_ = 360 nm; λ_em_ = 460nm) in a micro-titre fluorescence plate reader (SpectraMax M5, Molecular Devices) in kinetic mode at an interval of 9 s for 5–10 min.

### Temperature-dependent activity profile and thermal stability of InpA

The temperature-dependent activity profile was determined by pre-incubating the enzyme for 10 min in 0.1M Tris–HCl buffer, pH 7.5, containing 2 mM DTT, at temperatures in the range 4 to 60 °C, and then assaying activity at these temperatures using azocoll as substrate. To determine the thermal stability of InpA, the enzyme was pre-incubated for 30 min at temperatures in the above range, and in the above buffer, followed by assay with Z-Arg-Arg-AMC at 37 °C.

### Incubations of InpA with albumin

InpA was incubated with apo-and haemalbumin at 37 °C at 1:4 enzyme to substrate ratio, at pH 5.5 (in 0.06 M potassium hydrogen phthalate), pH 6.0 (0.2 M phosphate buffer, pH 6.0) or pH 7.5 (0.14 M NaCl, 0.1M Tris–HCl). Incubations were terminated by incubation for 30 min at 20 °C after addition of 0.5 mM E-64 to inhibit InpA activity, and then sampled for SDS-PAGE on 10 % gels under non-reducing conditions. For some experiments, to examine the effects of reducing conditions, buffers were supplemented with 3 mM DTT. For MALDI-TOF-MS analysis incubations were done in 10 mM Tris–HCl buffer (pH 7.5) with or without 3 mM DTT.

### Haemalbumin

Haemalbumin was prepared by incubating a 100 μM stock solution of bovine albumin (Sigma-Aldrich; product A-8763) at 37 °C in 0.14 M NaCl, 0.1 M Tris–HCl, pH 7.5, with iron(III) protoporphyrin IX at a 1:1 protein to haem molar ratio [31].

### Haem release from immobilised haemalbumin (agarose)

Bovine albumin-agarose beads (Sigma-Aldrich; product A3790) were washed in 0.14 M NaCl, 0.1M Tris–HCl, pH 7.5, until no unconjugated free albumin could be detected by UV absorbance at 280 nm in the eluate. The albumin was then complexed with haem (albumin to haem molar ratio 1:1) by gentle agitation at 4 °C for 18 h, and the beads (45 μM with respect to albumin) were incubated with gentle agitation with or without 2 μM InpA and with or without 3 mM DTT in 0.14 M NaCl, 0.1M Tris–HCl, pH 7.5 at 37 °C. Aliquots of the whole incubation mixture were periodically removed, having first been thoroughly mixed to ensure homogeneity. The samples were then centrifuged (5,000 g for 1 min at 20 °C) and the supernatants were removed and assayed for haem release by the pyridine haemochromogen method [53].

### Fluorescence monitoring of structural changes to albumin

Fluorescence emission of 16 μM albumin in the 310–410 nm range was measured at pH 7.5 in the presence of DTT (up to 100 μM), employing a Flexstation 3 Benchtop (Molecular Devices) multi-mode microplate reader using λ_ex_ = 295 nm [32, 33].

### SDS-PAGE

SDS-PAGE was carried out as previously described on either 10 or 12 % polyacrylamide gels [54]. Where appropriate, samples were solubilised at 100 °C for 5 min in reducing application buffer. For visualisation of protein-bound haem, samples were solubilised at 37 °C for 1 h in non-reducing application buffer and the gels were stained with tetramethylbenzidine-H_2_O_2_ (TMB) and then counter stained with Coomassie brilliant blue G250 (CBB) [13]. Concentrations of protein for SDS-PAGE were determined using the Bradford method.

### Densitometry

Densitometry was carried out on CBB- and TMB-H_2_O_2_-stained bands using UVIband gel analysis software (UVItech Ltd., Cambridge, UK) after digital image capture using a UMax Powerlook 1000 flatbed transmission scanner. Digital pixel volumes of CBB- and TMB-H_2_O_2_-stained bands were used as indirect measures of protein and haem loss from a sample (calculated as a percentage of the time zero sample).

### Determination of pH activity profile of InpA using azoalbumin

The activity profile of InpA over the pH range 3 to 10 was determined with azoalbumin, using 0.1 M sodium acetate for pH 3–5.5, 0.1 M sodium phosphate for pH 5.5-7.0, and 0.1 M Tris–HCl for pH 7–10. Azoalbumin (3 % w/v) was incubated with enzyme (final protein concentration 50 μg ml^−1^) in the above buffers together with freshly added DTT (3 mM) at 37 °C with shaking for 1 h. Subsequently, the protease activity was inhibited by mixing with two volumes of 10 % (w/v) ice-cold TCA. After 10 min, the insoluble protein was pelleted by centrifugation (10,000 × g for 10 min), and released azo dye was measured at 440 nm.

### MALDI-TOF-mass spectrometry

This was carried out using a Micromass M@LDI mass spectrometer, using an α-cyano-4-hydroxy-cinnamic acid matrix (Sigma-Aldrich). The spectra were recorded in the positive ion mode and the mass range scanned was 800 to 4000 Daltons. The spectrometer was calibrated using a mixture of authentic peptide samples. The observed peptide masses were compared to those predicted by theoretical cleavage using the PeptideMass program (ExPASy).

## Abbreviations

InpA: Interpain A
CBB: Coomassie brilliant blue
DTT: Dithiothreitol
SDS-PAGE: Sodium dodecyl sulphate polyacrylamide electrophoresis
